# Seroprevalence and trends of hepatitis B virus, hepatitis C virus and human immunodeficiency virus in Syrian blood donors at Damascus University Blood Center between 2004 and 2021

**DOI:** 10.3389/fpubh.2023.1174638

**Published:** 2023-06-01

**Authors:** Alia Alassad, Mhd Jawad Al Rahwanji, Amal Yousfan, Sally Al Moualem, Arwa Farhat, Lama A. Youssef

**Affiliations:** ^1^Damascus University Blood Center, Damascus University, Damascus, Syria; ^2^Department of Artificial Intelligence, Faculty of Information Technology Engineering, Damascus University, Damascus, Syria; ^3^Department of Computer Science, Saarland University, Saarbrücken, Germany; ^4^Department of Pharmaceutics & Pharmaceutical Technology, Faculty of Pharmacy, Damascus University, Damascus, Syria; ^5^Department of Pharmaceutics and Pharmaceutical Technology, Pharmacy College, Al Andalus University for Medical Sciences, Tartus, Syria; ^6^Department of Biochemistry and Microbiology, Faculty of Pharmacy, Damascus University, Damascus, Syria; ^7^Program of Clinical and Hospital Pharmacy, Department of Pharmaceutics & Pharmaceutical Technology, Faculty of Pharmacy, Damascus University, Damascus, Syria

**Keywords:** transfusion-transmitted viral infections, hepatitis B virus, hepatitis C virus, human immunodeficiency virus, blood donors, Syria

## Abstract

**Introduction:**

Seroprevalence of transfusion-transmitted viral infections (TTVIs) is a valuable indicator for assessing blood safety, population health and health system performance in the times of peace and conflicts. Only scarce information is available on the impact of the decade-long violent conflict on the prevalence of TTVIs in Syria. Moreover, hepatitis B vaccine was introduced to the national vaccination program in 1993; however, no data is available on the vaccine effectiveness.

**Methods:**

In this retrospective cross-sectional study, we compiled the screening results for major TTVIs, namely hepatitis B virus (HBV), hepatitis C virus (HCV), and human immuno-deficiency virus (HIV), of volunteer donors at Damascus University Blood Center from May 2004 to October 2021. Prevalence was expressed in percentages for the entire study group and subgroups. Chi-square test and linear regression were used to examine the differences and describe trends in prevalence, respectively, based on demographic characteristics (i.e., age and gender) and time. *P*-value of <0.005 was considered statistically significant.

**Results:**

Of the total 307,774 donors (82.27% males, median age 27 years), 5,929 (1.93%) had serological evidence of at least one TTVI, and 26 (0.0085%) had multiple infections. The lowest prevalence (1.09%) was detected in donors aged 18–25 years old, and a higher prevalence (2.05%) was evident in males in comparison with females (1.38%). The seroprevalence of HBV, HCV, and HIV was 1.18, 0.52, and 0.23%, respectively. Trend analyses revealed a significant regression in HBV and HIV prevalence from 2011 to 2021. HBV seropositivity depicted a temporal decline by ~80%, from 0.79% in 2011 to 0.16% in 2021 in those born in 1993 and thereafter.

**Discussion:**

The seroprevalence of HBV, HIV, and to a lesser extent HCV dropped over the study 18-year period. Possible explanations may include implementation of the HBV vaccine, robust national health system, conservative sociocultural values, and isolation.

## 1. Introduction

Blood transfusion is a vital service provided by healthcare systems worldwide. It could be a life-saving or health-improving medical intervention for millions of patients around the world every year.

Indications of blood transfusion in low- and middle-income countries, encompass symptomatic anemia, acute blood loss (such as pregnancy-related complications), as well as hemoglobinopathies, mainly sickle cell disease (SCD) and thalassemia.

Transfusion in SCD patients can be either episodic in acute care settings or part of life-long management. Whereas, regular long-term blood transfusion is the mainstay of the clinical management to maintain pre-transfusion hemoglobin concentrations above 95–105 g/L in β-Thalassemia major (BTM) (1–3).

These hereditary hemoglobinopathies are prevalent in countries of the Mediterranean basin across Africa and the Middle East, being almost all in the so-called “thalassemic belt.” Blood banks in these countries pledge to provide adequate and safe blood supply in response to the increased demands for blood and blood products while maintaining appropriate hemovigilance (4, 5).

Blood transfusion is generally considered safe, as blood safety is a recognized priority in blood banks worldwide. However, transfusion may yet hold some risks and complications. One of the most serious risks to say is that of transfusion-transmitted viral infections (TTVIs) (3, 5). Of the TTVI-causing pathogens, hepatitis B virus (HBV), hepatitis C virus (HCV), and human immunodeficiency virus (HIV) are of major concern.

Both HBV and HCV viruses can cause acute and chronic infections and potentially lead to the development of serious liver problems, including liver failure, cirrhosis and hepatocellular carcinoma, which ultimately cause high mortality. HBV and HCV could be transmitted horizontally via exposure to infected blood from sources, such as unsafe injection practices, unscreened blood transfusions, unsafe health care, and injection drug use, and via sexual route; or vertically from mother to child at birth, though the last two routes are less common for HCV (6, 7).

According to the World Health Organization's (WHO) estimates, the global prevalence of chronic hepatitis B and C infections in 2019 reached 296 and 58 million people, respectively, with almost an identical incidence of 1.5 million new cases. In 2019, viral hepatitis caused ~1.1 million deaths, with 820,000 and 290,000 deaths attributed to hepatitis B and C, respectively. Cirrhosis and hepatocellular carcinoma are the leading causes of death (6, 7). Whereas, an effective and safe recombinant vaccine has been available since 1986 for prophylaxis against HBV (8), HCV genetic diversity, limited models for testing vaccines, and incomplete understanding of protective immune responses have hindered the efforts to develop an effective vaccination strategy against HCV (9).

HIV continues to be a crucial universal public health issue, having claimed 680,000 lives due to its complications in 2020. Although new HIV infections have decreased by 52% since 1997, the global burden of the disease is still high; with over 36 million living with HIV and ~1.5 million new cases of HIV reported in 2020 (10–12). HIV shares the same transmission modes of HBV and HCV, including unsafe sex, blood transfusions, and sharing drug injection equipment (i.e., needles and syringes), in addition to the vertical perinatal transmission from mother-to-child during pregnancy, delivery or breastfeeding (11, 13–16).

The majority of studies conducted to assess the prevalence of HBV and HCV in Syria were constrained to high-risk groups, primarily patients with hematological diseases who receive periodic blood transfusions (17) and hemodialysis patients (18), and limited published data exist on the prevalence of viral hepatitis in the Syrian general population. Moreover, only a paucity of studies investigated the impact of introducing the HBV vaccine to the Syrian national childhood vaccination program in 1993 on HBV epidemiology (19).

According to a serological survey conducted in 2004 by the Syrian Ministry of Health (MoH) in collaboration with the Central Bureau of Statistics, the seroprevalence of hepatitis B and C was 5.6 and 2.8%, respectively, with a clear inter-regional variation. On the other hand, the prevalence of seropositivity was lower among blood donors as reported by the Syrian MoH during the period from 2003 to 2014, with a similar regional discrepancy to that previously reported in the general population. In 2014, the recorded prevalence rates of HBV and HCV at the national blood banks were 1.1 and 0.4%, respectively (19). A recent study carried out in the coastal city of Tartus reported seropositivity rates in local blood donors of 0.56 and 0.48% for HBV and HCV, respectively (20).

Noticeably, a meta-analysis and systematic review that characterized and analyzed all available published and unpublished records of HCV epidemiology in countries of the Fertile Crescent region revealed a prevalence rate of 47.4% (range: 21.0–75.0%; 95% CI: 32.5–62.5%) of hepatitis C in high-risk population(s) (hemodialysis and hemophilia patients and drug users), but a lower rate of 0.4% (range: 0.3–0.9%; 95% CI: 0.4–0.5%) in blood donors at national blood banks (21).

Syria is considered a country with low endemicity of HIV (< 0.1% among the general population), with an incidence to prevalence ratio ranging between 5.0 and 9.99 (11, 12, 22). According to the Global Burden of Disease, HIV ranks seventh among communicable diseases in causing fatalities in Syria and has a 30.22 disability-adjusted life years (DALYs) per 100,000 population (23). As claimed by the Syrian MoH, of the 1,141 HIV infection cases registered between 1987 and 2022, only 786 were Syrians (24). The majority of the existing epidemiological data on HIV is based on officially reported cases from routine screening of blood donors, which constitutes ~62% of all HIV tests, followed by premarital HIV testing (19%) introduced in 2010, and testing of Syrian emigrants (8.5%) and tests conducted at other health centers and clinics (10.5%) (22, 25).

To ensure blood safety, availability, and sustainability, the national program for blood supply in Syria adhere to the WHO recommendations on assessing donors' eligibilities and screening donated blood, using recommended serological assays with a sensitivity and specificity not less than 99.5%, such as enzyme immunoassay (EIA) and chemiluminescence microparticle immunoassay (CMIA) (17). All blood donations in Syrian blood banks are mandatorily screened for hepatitis B, by screening for hepatitis B surface antigen (HBsAg); hepatitis C by screening for HCV antibodies (anti-HCV); HIV-1/2 by screening for a combination of HIV antigen-antibody.

A comprehensive assessment of TTVIs prevalence and trends among Syrian blood donors may contribute to a more effective national overall infection control strategy, as it reflects the prevalence and trends of these infections in the general population, nevertheless blood donors are generally regarded as a healthier population.

In this study, we aimed at assessing the prevalence rates and trends of serological markers of the classic TTVIs; namely HBV, HCV, and HIV, among blood donors at Damascus University Blood Center between 2004 and 2021. Negative correlation between hepatitis B vaccination coverage and HBsAg prevalence has been reported in epidemiological studies from multiple countries, including those with high as well as low endemicity of HBV. However, data are lacking on the impact of introducing the HBV vaccine to the national infant vaccination program on HBV prevalence. Data collected on the temporal trend(s) of HBV prevalence in Syrian blood donors over 18 years (from 2004 to 2021) are expected to quantitatively elucidate the effectiveness of incorporating hepatitis B vaccine in the national vaccination program.

## 2. Materials and methods

### 2.1. Study design

We comprehensively reviewed the records of blood donors at Damascus University Blood Center from May 2004 to October 2021. Damascus University Blood Center was established in 1998 for research, training and service provision and is one of Syria's busiest blood banks. On average, 25,557 units of blood are collected annually, mainly from undergraduate and graduate students, faculty members, and volunteers in blood donation campaigns; and the majority of the collected blood bags are provided to tertiary referral hospitals.

### 2.2. Serological investigations

The recommended standard measures were used for blood donor recruitment, selection, testing and deferral. Blood donors were volunteers, and their eligibility requirements for donation were determined based on their medical history and physical and hematological examinations prior to blood donation. For allogeneic use, all collected bags underwent screening for the ABO blood type, rhesus (Rh) status, and TTVI markers, such as HBsAg and anti-HCV and HIV antibodies/antigens, as well as alanine aminotransferase (ALT) to safeguard population health. Samples were tested using SURASE B-96 (TMB) for the *in vitro* qualitative detection of Hepatitis II surface antigen (HBsAg), NANBASE C-96, V4.0 for the *in vitro* qualitative detection of antibody to Hepatitis C virus (anti-HCV) and GB HIV Ag-Ab COMB for *in vitro* qualitative detection and screening of HIV infection (General Biologicals Corporation, Taiwan). Microplates were read using 800 TS Absorbance Reader (BioTek, USA). When a test returned positive, two additional samples were collected from the questionable blood bag to confirm positivity. The additional confirmatory tests were done using the same make of the test kit. Any blood sample tested positive for TTVIs, or had abnormal levels of ALT, was discarded and the donor was permanently deferred as well as referred to appropriate clinics for treatment.

### 2.3. Data collection and statistical analysis

The data pertaining to 367,019 individuals was obtained from the electronic database generated by the Delphyn software (Version 6.5.0; Hemasoft) used at Damascus University Blood Center. Data was extracted using SQL queries from the underlying InterBase database and analyzed using Python and Python packages namely Scipy (Version 1.7.3) which was used for statistical analysis. A total of 59,245 individuals were deferred over the 18 years due to having low hematocrit (Hct) and/or hemoglobin (Hb), suboptimal weight or some other undocumented medical condition. After being checked for completeness, the socio-demographic variables (gender and age) and serological test results of the remaining 307,774 (~84%) donors were analyzed. The overall prevalence of each transfusion-transmitted viral infection was calculated by dividing the number of donors with a confirmed-positive particular TTVI serologic marker by the total number of blood donors and expressed as percentages in the study period. Likewise, the annual prevalence of each TTVI was calculated by dividing the number of donors with confirmed-positive particular TTVI serologic marker in a year by the total number of blood donors in that year, for males and females and within five age groups; 18–25, 26–35, 36–45, 46–55, and 56–65 years old. Chi-square test was conducted to examine the differences in prevalence between males vs. females, the different age groups and year-to-year variation throughout the study period from 2004 to 2021. The odds ratio was subsequently used to quantify the association perceived when Chi-square test reported significance. Linear regression was used to detect trends in prevalence and incidence for HBV, HCV and HIV over time (between 2004 and 2021). *P*-values of ≤ 0.005 were considered statistically significant.

### 2.4. Ethical considerations

The study was conducted after obtaining ethical approval from the Faculty of Pharmacy at Damascus University. Additionally, endorsement was also obtained from the administration of the Damascus University Blood Center. The data was analyzed confidentially and personal identifiers were blinded to ensure anonymity. Since the study was conducted retrospectively, an informed consent was not sought from study participants.

## 3. Results

### 3.1. Socio-demographic characteristics of the study population

**Figure 1:**
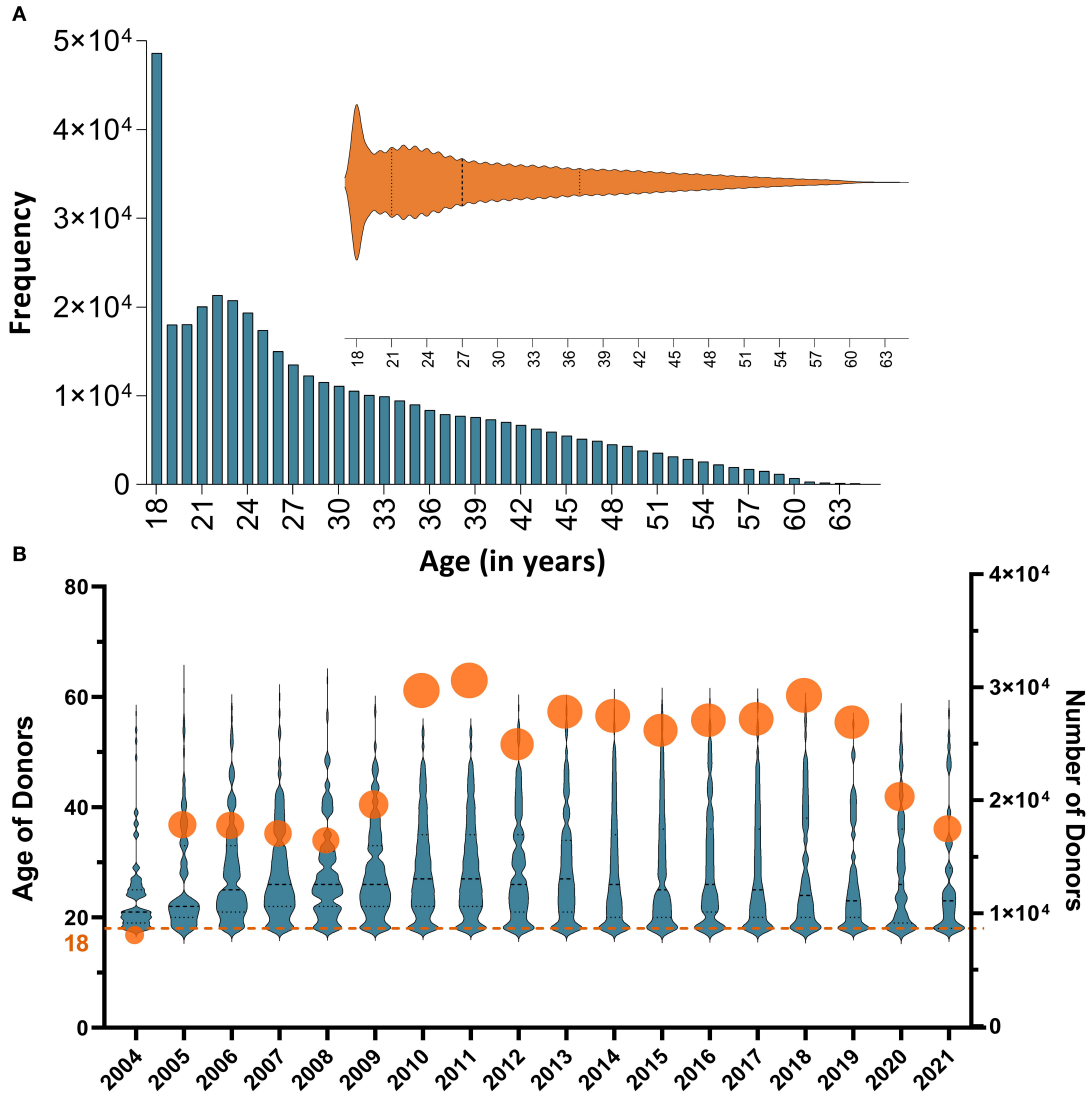
**(A)** A histogram representation of the distribution of the study donor' ages. Approximately, 45% of the donors were ≤ 25 with the most frequent age being 18 year; **(B)** Annual numbers of donors and distributions of their ages throughout the study period (from 2004 to 2021). The summary statistics and the (probability) density of donors' age per year are illustrated using violin plots; all medians hover around 30 years of age. Numbers of participants in each year are also shown using circles (the size of the circle depicts the variability in the number of donors); annual numbers of donors varied between <4 × 10^4^ in 2004 and 1.4 × 10^5^ in 2011.

A total of 307,774 blood donors fulfilled the eligibility requirements for blood donation and had complete records in the database; 253,219 (82.27%) were males and 54,555 (17.73%) were females. Donor age ranged from 18 to 63 years, with a median age of 27 years. Approximately, 45% of the donors were 18–25 years old with the majority (11.8%) being 18 years old (Figure 1). Nearly 81% of donors gave one donation, whereas 19% gave multiple donations throughout the study period.

### 3.2. Trends of seroprevalence of transfusion-transmitted viral infections

**Figure 2:**
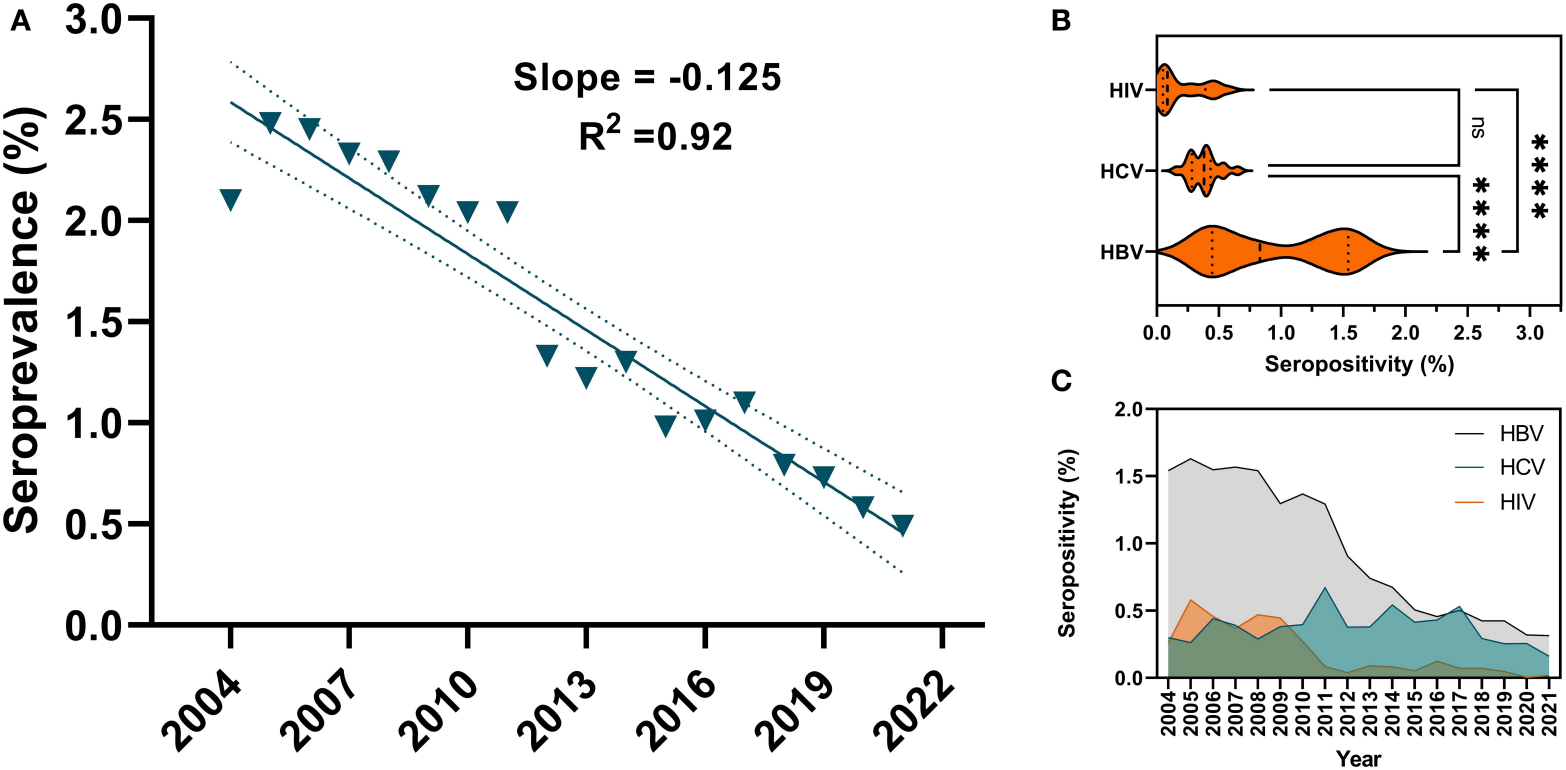
Linear regression of temporal trends of cumulative TTVIs seropositivity and general seroprevalence of HBV, HCV and HIV at Damascus University Blood Center from 2004 to 2021. **(A)** Cumulative TTVIs linear regression showed a steady decrease in seroprevalence throughout the 18-year period of the study. **(B)** A Violin plot depicted the distributions of HBV, HCV and HIV' seropositivity data and their densities, and revealed a wider range of prevalence variability for HBV in comparison with HCV and HIV, which exhibited minimal changes across the study period. Analysis of Variance (ANOVA) showed that HBV was the most prevalent TTVI (*P*-value < 0.0001). **(C)** A triple comparison of temporal seropositivity of HBV vs. HCV vs. HIV. HCV positivity remained relatively unchanged throughout the 18 years, whereas seropositivities of HBV and HIV started to drop in 2012 and 2011, respectively.

Serological evidence of at least one infection was confirmed in 5,929 (1.93%) of the total blood donors. Table 1 summarizes annual seropositivities of HIV, HBV, and HCV in blood donors at Damascus University Blood Center from 2004 to 2021. HBV was the most prevalent (3,644, 1.18%) TTVI throughout the 18 years, followed by HCV (1609, 0.52%) and HIV (702, 0.23%) (Figures 2B, C). In donors ≤ 25 years old, there was a downward tendency in TTVIs prevalence in comparison that in those >25 years old (OR = 0.62, 95% CI: 0.59–0.66, *P*-value < 0.0001). A higher prevalence (5201, 2.05%) was evident in males in comparison with that in females (754, 1.38%) (OR = 1.49, 95% CI: 1.38–1.61, *P*-value < 0.0001). Over the 18-year period, the frequency of TTVIs showed a proportional drop over years (β = −0.13, 95% CI: −0.15 to −0.11, *R*^2^ = 0.92, *P*-value < 0.0001) (Figure 2A) and Chi-square test demonstrated a significant decrease in TTVI trends since 2011 (OR = 0.48, 95% CI: 0.41–0.55, *P*-value < 0.0001) (Table 2).

**Table 1:** Annual numbers of donations, acceptance rates and seropositivities of HIV, HBV, and HCV in blood donors at Damascus University Blood Center from 2004 to 2021.

**Year**	**Number of donors**	**Accepted donors *n* (%)**	**Seropositivity (%)**			
			**HBV**	**HCV**	**HIV**	**Cumulative TTVIs**
2004	8,213	8,063 (98.2)	1.54	0.30	0.26	2.10
2005	18,930	17,830 (94.2)	1.64	0.26	0.58	2.48
2006	19,227	17,763 (92.4)	1.55	0.44	0.46	2.45
2007	18,390	17,047 (92.7)	1.57	0.39	0.37	2.33
2008	17,528	16,440 (93.8)	1.54	0.29	0.47	2.29
2009	21,235	19,611 (92.4)	1.29	0.38	0.45	2.12
2010	31,694	29,700 (93.7)	1.37	0.40	0.27	2.04
2011	32,524	30,578 (94.1)	1.30	0.65	0.08	2.04
2012	26,749	24,948 (93.1)	0.91	0.38	0.04	1.33
2013	30,023	27,828 (92.7)	0.75	0.38	0.09	1.22
2014	29,741	27,453 (92.3)	0.68	0.54	0.08	1.30
2015	28,335	26,182 (92.4)	0.51	0.42	0.05	0.98
2016	29,401	27,071 (92.1)	0.45	0.43	0.13	1.01
2017	29,515	27,190 (92.1)	0.50	0.53	0.07	1.10
2018	31,736	29,270 (92.2)	0.43	0.29	0.07	0.79
2019	29,205	26,904 (92.1)	0.42	0.26	0.05	0.73
2020	21,803	20,315 (93.2)	0.32	0.26	0	0.58
2021	18,428	17,466 (94.8)	0.31	0.16	0.02	0.49

**Table 2:** Analyses of the associations between seroprevalence of transfusion-transmitted viral infections and the blood donors' demographic characteristics (age and gender) and time (years) between May 2004 and October 2021.

**Infection**	**Characteristics**	**Prevalence**			
		**(%)**	**Odds ratio**	**95% CI**	**Chi-square** ***P*****-value**
**HBV**	**Gender**				
	Males	89.41	1.83	1.64–2.03	< 0.0001
	Females	10.59	1		
	**Age group (years old)**				
	18–65				< 0.0001
	18–25	27.99	0.48	0.45–0.52	< 0.0001
	26–65	72.01	1		
	**Years**				
	Before 2012	49.69	1		< 0.0001
	Since 2012	50.31	0.37	0.3–0.45	
	Males (2005–2011)	92.73	1.79	1.16–2.77	0.0061
	Females (2005–2011)	7.27	1		
	Males (since 2012)	84.13			0.8207
			–		
	Females (since 2012)	15.87			
**HCV**	**Gender**				
	Males	83.84			0.1
			–		
	Females	16.16			
	**Age group (years old)**				
	18–65				0.0002
	18–25	41.08	0.87	0.78–0.96	0.0045
	26–65	58.92	1		
	**Years**				
	Before 2011	27.26			0.54
			–		
	Since 2011	72.74		
	Before 2018	85.3	1		0.0005
	Since 2018	14.7	0.57	0.41–0.78	
**HIV**	**Gender**				
	Males	84.62			0.11
			–		
	Females	15.38			
	**Age group (years old)**				
	18–65		–		0.29
	18–25	44.73			0.97
			–		
	26–65	55.27			
	**Years**				
	Before 2011	68.83	1		< 0.0001
	Since 2011	31.17	0.24	0.14 to 0.4	
**Cumulative TTVIs**	**Gender**				
	Males	87.34	1.24	1.15 to 1.34	< 0.0001
	Females	12.66	1		
	**Age group (years old)**				
	18–65				< 0.0001
	18–25	33.5	0.62	0.59–0.66	< 0.0001
	26–65	66.5	1		
	**Years**				
	Before 2011	46.23	1		< 0.0001
	Since 2011	53.77	0.48	0.41–0.56	

Of the 307,774 donors, only 26 (0.0085%) (25 males and 1 female) donors were co-infected with two infections. The majority 18 (69.23%) was attributed to HBV-HCV co-infection, six (23.07%) had HBV-HIV co-infection and two (7.69%) donors had HCV-HIV co-infections.

### 3.3. Prevalence of HBV and associated risk factors

**Figure 3:**
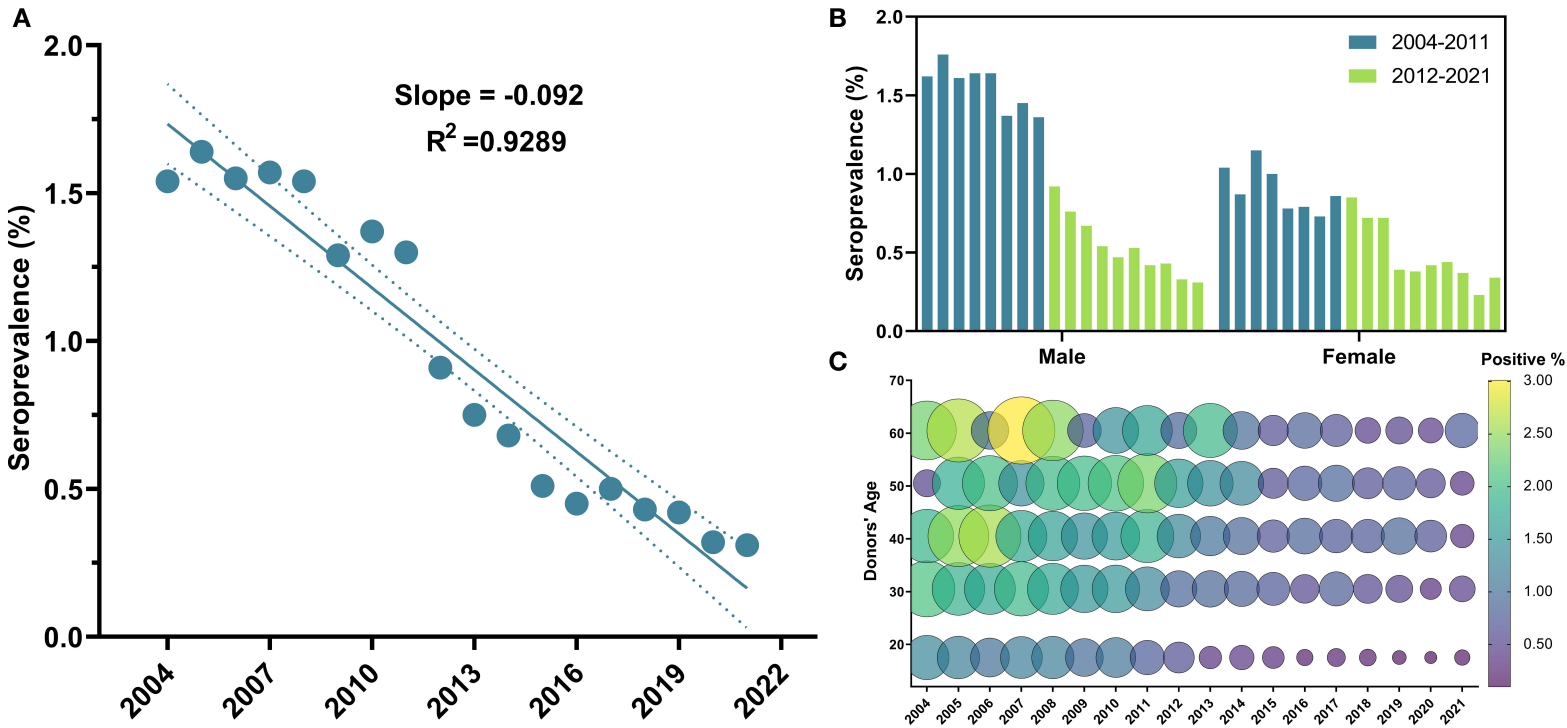
Temporal trends of HBV general seroprevalence, between male and female blood donors and across age groups from 2004 to 2021. **(A)** HBV depicted a significant linear regression throughout the 18 years of the study; **(B)** HBV positivity was significantly higher in males than females, and the aforementioned drop in HBV positivity can be clearly perceived in males who constituted that majority of the study population; **(C)** Bubble plot of HBV seropositivity across age groups from 2004 to 2021. At all-time points, HBV seropositivity was higher in the older age groups. Starting in 2012, an apparent decline in HBV seropositivity can be depicted in donors under 25 years old.

The overall sero-prevalence of HBV was 3,644 (1.18%), with males having higher prevalence (1.28%) compared to females (0.7%) (OR = 1.83, 95% CI: 1.64–2.03, *P*-value < 0.0001). Annual trends of HBV seropositivity per 100 donors exhibited a significant linear decrease (β = −0.092, 95% CI: −0.11 to −0.08, *R*^2^ = 0.93, *P*-value < 0.0001) (Figure 3A). The lowest seroprevalence was in blood donors in the age group of 18–25 years old compared to the rest of the population (OR = 0.48, 95% CI: 0.45–0.52, *P*-value < 0.0001) (Table 2 and Figure 3C) (Supplementary Figure S1). Additionally, a significant drop in prevalence among blood donors can be perceived since 2012 (OR = 0.37, 95% CI: 0.3–0.45, *P*-value < 0.0001) (Table 2). Males to females' prevalence discrepancy was evident from 2004 until 2011; as males had almost twice higher prevalence than females (OR = 1.79, 95% CI: 1.16–2.77, *P*-value = 0.0061), however the probability of being seropositive was almost equalized since 2012 (*P*-value = 0.82) (Table 2 and Figure 3B).

### 3.4. Prevalence of HCV and associated risk factors

**Figure 4:**
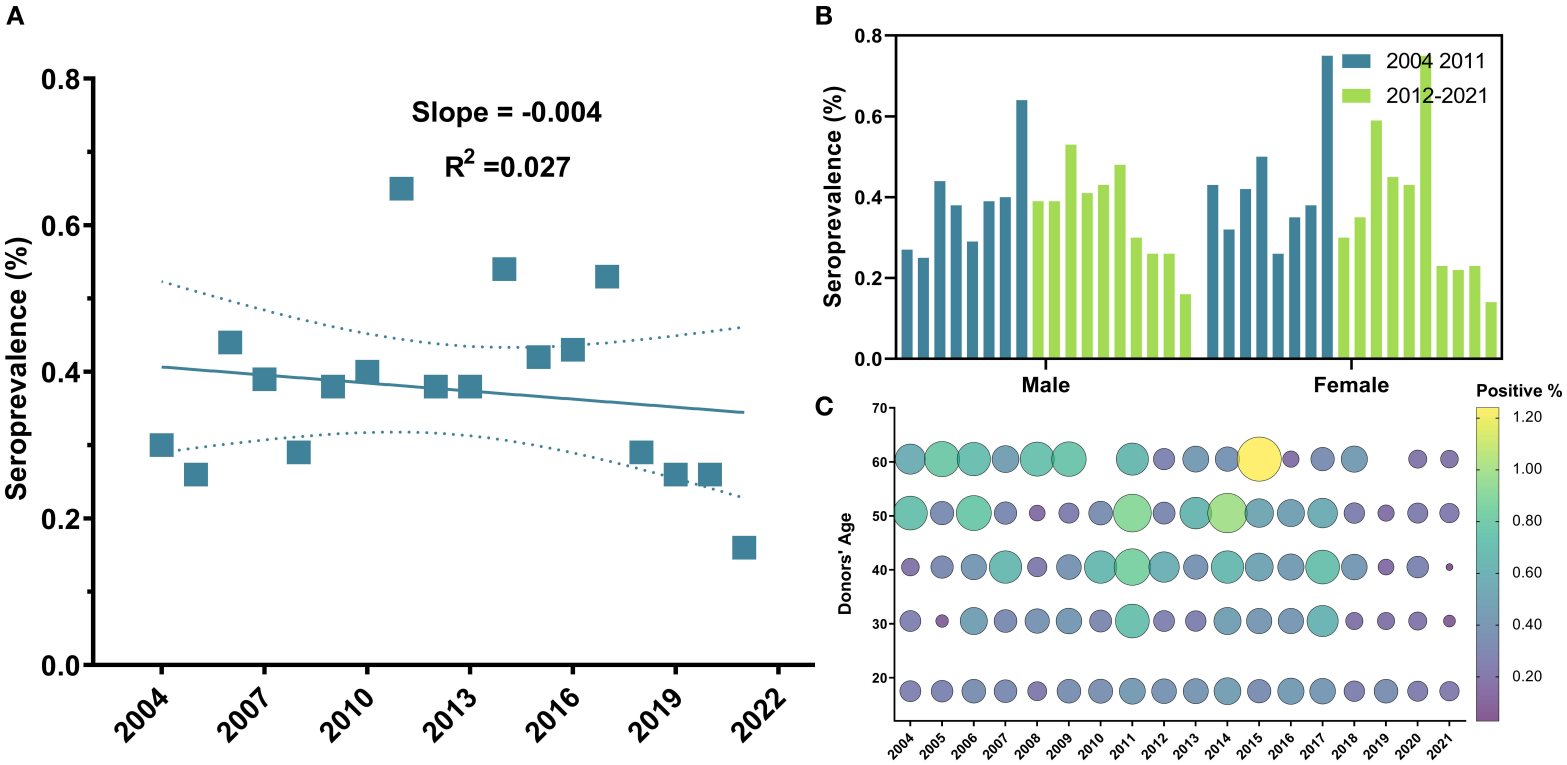
Temporal trends of HCV general seroprevalence, between male and female blood donors and across age groups from 2004 to 2021. **(A)** Sequential seroprevalence of HCV remained relatively unchanged. **(B)** No significant difference in prevalence was observed between males and females along study period; and **(C)** Bubble plot of HCV seropositivity across age groups from 2004 to 2021. HCV positivity remained unchanged throughout the years of the study and was slightly higher in older age groups.

The overall seroprevalence of HCV among blood donors was 1,609 (0.52%). However, this prevalence fluctuated over years and annual trends did not show a significant linear decrease (*R*^2^ = 0.03, *P*-value = 0.51) (Figure 4A). On the contrary to HBV, HCV prevalence did not depict any significant decrease in prevalence since 2012 (*P*-value = 0.54). Instead, a remarkable drop in prevalence was observed starting in 2018 (OR = 0.57, 95% CI: 0.41–0.78, *P*-value = 0.0005) (Table 2). Donors in the age group 18–25 years old were less likely to have HCV infection compared to other age groups (OR = 0.87, 95% CI: 0.78–0.96, *P*-value < 0.0045) (Table 2 and Figure 4C) (Supplementary Figure S1), but no significant difference in prevalence was observed between male (0.53%) and female donors (0.47%) (*P*-value = 0.1) (Table 2 and Figure 4B).

### 3.5. Prevalence of HIV and associated risk factors

**Figure 5:**
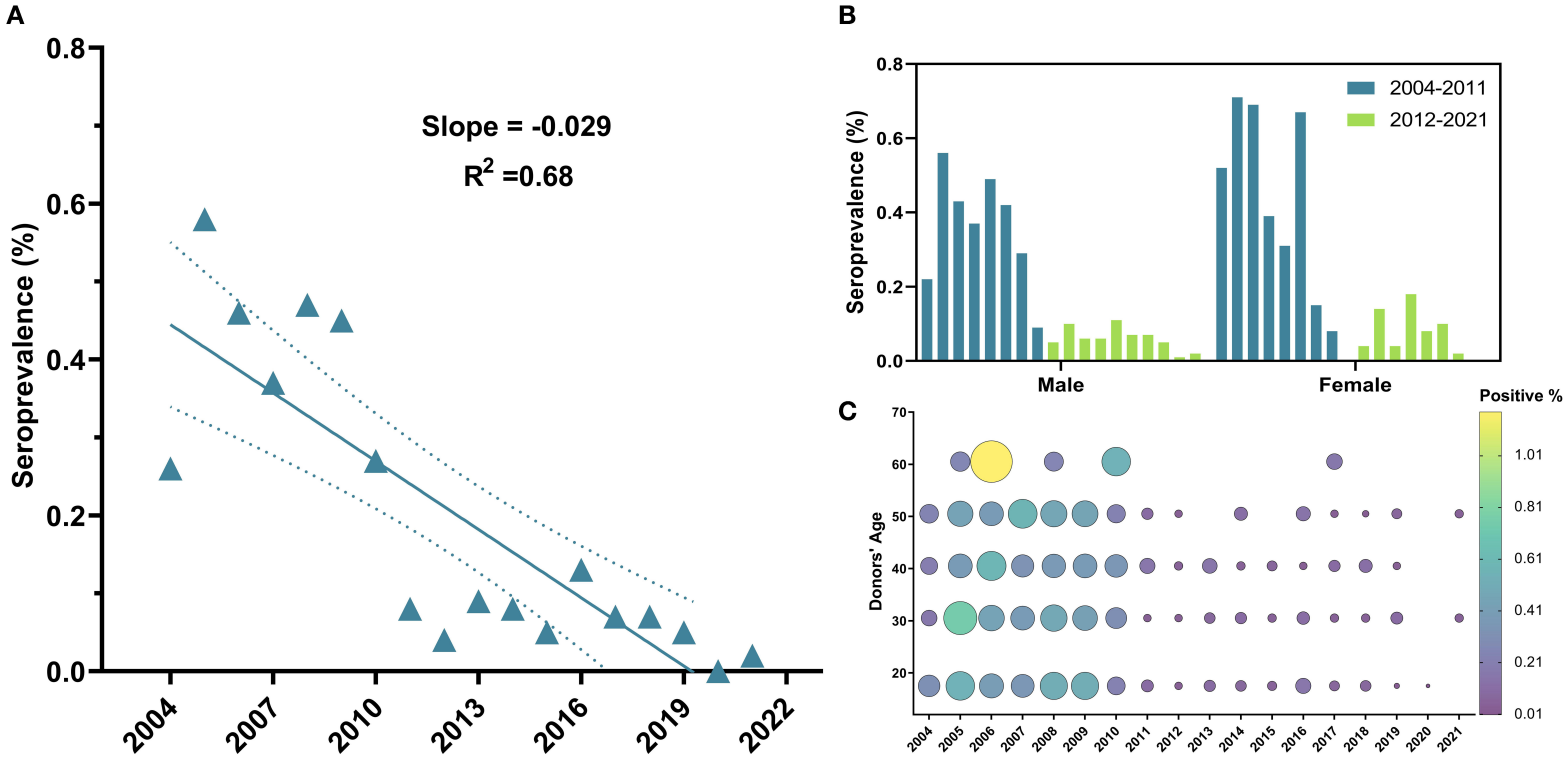
Temporal trends of HIV general seroprevalence, between male and female blood donors and across age groups from 2004 to 2021. **(A)** HIV exhibited a downward but less linear trend compared to HBV. **(B)** No significant difference in prevalence was observed between males and females along study period and drop in HIV can be clearly perceived in both males and females since 2011. **(C)** Bubble plot of HIV seropositivity across age groups from 2004 to 2021. HIV positivity was almost spread out equally across age groups and started to drop in 2011.

The overall seroprevalence of HIV infection among blood donors was 702 (0.23%). Temporal trends of HIV prevalence exhibited a declining tendency (β = −0.03, 95% CI: −0.04 to −0.02, *R*^2^ = 0.68, *P*-value < 0.0001) (Figure 5A); and a significant drop in prevalence was observed since 2011, with a steep reduction of 70.37% in HIV prevalence from 2010 (0.27%) to 2011 (0.08%) (Table 1) and throughout the following years until 2021 (OR = 0.24, 95% CI: 0.14–0.40, *P*-value < 0.0001) (Table 2). Noteworthy, no significant difference in regards to HIV prevalence was found between the five age groups (*P*-value =0.97) (Table 2 and Figure 5C) (Supplementary Figure S1) or between males (0.23%) vs. females (0.2%) (*P*-value = 0.11) (Table 2 and Figure 5B).

## 4. Discussion

Blood banks are valuable sources of epidemiological data, especially in developing countries and during humanitarian crises. Assessing and monitoring prevalence trends of TTVIs in blood donors provide an opportunity to evaluate the safety of blood supply and the effectiveness of donor deferral criteria and implemented hemovigilance protocols. Moreover, it may portray the spread of TTVIs in the general population as well. The WHO Global Database on Blood Safety (GDBS) periodically publishes statistics on blood donations and acquired blood-borne infections worldwide. Unfortunately, these statistical estimates lack data on TTVI prevalence in Syria (5, 26). Therefore, our study aimed at filling an epidemiological data gap regarding TTVIs prevalence in Syrian blood donors spanning 18 years; from 2004 to 2011 (pre-conflict) to 2012–2021 (during the conflict).

Present-day Damascus is a modern metropolis that offers employment and education opportunities to native residents as well as to comers from rural areas of the country. Furthermore, Damascus and its countryside have housed large numbers of displaced Syrians during the 10-year crisis. Moreover, Damascus University is considered the largest and most diverse university in Syria. Taken together, this admixture has made Damascus a representative microcosm of Syria and rendered the Damascus University Blood Center a mini-model simulating Syrian blood banking.

Our comprehensive analysis of blood donors' records at the Damascus University Blood Center revealed that females constituted only 17.73% of all donors; a percentage close to that reported from a similar study in Tartus (17.69%) (20), and which is nearly half of the global percentage (33%). On the other hand, when compared with Eastern Mediterranean countries, Syria ranks among the upper-middle countries in terms of women's participation in blood donations, as the region has a median of 6% female blood donors with substantial between-country differences (range from 0.4 to 31%). Besides, the Syrian gender blood donation profile is close to that of some other Arab countries such as Lebanon (19.96%), Egypt (19%) and Tunisia (16%) (5).

Literature explained the gender participation gap by many factors, some of which are physiological factors such as menstruation, lactation, pregnancy, lower hemoglobin levels, and higher susceptibility to vasovagal events; alongside other factors that include prejudices and socio-cultural aspects, which may have higher impact in developing countries (27–29).

GDBS data indicate that approximately half of donations worldwide were given by donors aged 25 to 44 years, however, a higher percentages of younger people (aged < 24 years) donate blood in low- and middle-income countries (38% and 28–47%, respectively) in comparison with 25% in high-income countries (5). Comparably, ~44.61% of all donors at Damascus University Blood Center were ≤ 25 years with median and mode ages of 27 and 18 years, respectively. These percentages are representative of the Syrian demographic structure, as the median age of the Syrian population is 20.2 years (30), and as a large percentage of Syrians are ≤ 30 years old (23, 31). Furthermore, nearly 81% of donors gave one donation, which contributes to a reduced estimation error and makes our sample population more representative of the general Syrian population.

Expectedly, and similar to other University affiliated blood banks, the majority of blood donors at Damascus University Blood Center are undergraduate students ranging in age from 18-year old (freshmen) to 22-year old (24-year old in medical schools) graduates applying for compulsory military service waiver and/or for driving license. During the 18-year period of the study, from 2004 to 2021, a general trend of seroprevalence decline was apparent for all TTVIs in blood donors at Damascus University Blood Center. This decrease in cumulative TTVI-seropositivity suggests a pattern that is likely influenced by behavioral and/or demographic changes that occurred in the general population and/or the donor population recruited to the national blood banks, and may also reflect successful educational and screening procedures leading to better deferral of high-risk individuals.

Our findings proved that HBV is the most prevalent blood-borne viral infection in Syrians. Screening of HBsAg in blood donors in the Eastern Mediterranean Region of Operations (EMRO) countries demonstrated a pooled prevalence of 2.03% (95% CI: 1.79–2.26), making HBV the most prevalent TTVI in the region despite its prevalence disparity (0.1–20%) in the region's countries (32). Our data revealed an HBV overall frequency of 1.18% in blood donors, which falls within the prevalence range reported by the WHO statistics in the EMRO region. Furthermore, we observed that the prevalence of HBV dropped from 2004 to 2021 by 80%. Statistics published by the Syrian Ministry of Health on TTVIs seropositivity in the local blood banks have also demonstrated a comparable drop in the HBV prevalence between 2003 and 2014 (19). Although the MoH's reported prevalence estimates were higher than ours, the calculated decline trend rate of 62% between 2004 and 2014 was comparable to the calculated 56% drop in our current study. This may be attributed to cultural differences and regional variations in levels of education across the Syrian geography and demography. The Syrian society is definitely heterogeneous in regards to cultures, traditions and level of awareness. This heterogeneity in social, cultural and educational factors is expected to determine the regional prevalence of infectious diseases. In agreement with our findings, a recent study conducted has reported a prevalence of 0.56% for HBV in blood donors at the coastal city of Tartus in 2017 (20). Similar to the capital Damascus, the Syrian coast has received a massive arrival of displaced people during the Syrian conflict, and the comparable frequencies of HBV seropositivity at Tartus blood transfusion center and Damascus University Blood Center may suggest homogeneity of blood donor populations at both centers.

The evident decline in HBV prevalence starting in 2012 could be mostly ascribed to the inclusion of HBV vaccine into the gratuitous and mandatory government-provided national immunization program in 1993 (19). Here, we report an apparent association between higher HBV prevalence and older ages ≥26, and additionally, starting in 2012 through 2021, remarkably lower HBV prevalence was obvious in blood donors of the youngest age group 18–25 years old, who are expected to have received the HBV vaccine (Figure 3C). This observation was consistent with results obtained from Blood Transfusion Center of Tartus (20) and among multi-transfused Patients with Hemoglobinopathy (17). Moreover, the deterioration of the immune system, termed immunosenescence, is related to aging, which makes older people more vulnerable to infections (33). Worth mentioning, the dropping in the coverage of the HBV vaccination during the Syrian crisis from 84% in 2010 to 42% in 2016 (34) may lead to a surge in infections in some territories of the country in the upcoming years. Hence, we believe that it is of extreme importance for the Syrian health authorities to regularly report the status of TTVIs, develop an infection-aware culture, and maintain and expand the HBV vaccination to reach 100% coverage of neonates and adolescents before they engage in high-risk practices.

Another possible explanation of the observed regression in HBV prevalence may come from the Syrian crisis itself that started in 2011 and led to the segmentation of the country. The north eastern rural region of Syria, where typically higher TTVIs seropositivity was reported prior to the conflict, was militarily and demographically segregated from the territories dominated by the Syrian regimen.

Disproportionate male to female seropositivity ratio was depicted for the overall TTVIs investigated and for HBV in particular. Our findings are in agreement with those of studies from Iran, Egypt and Ethiopia (35–38). This gender disparity might be partly due to the fact that males are ~1.5 times more likely to develop chronic HBV infection than females as a result of the slower plasma clearance rate of HBsAg in males compared to females. Moreover, some studies suggested a genetic determinant of disease development mainly regulated by sex hormones (28, 33, 35). Furthermore, the higher TTVIs prevalence in male donors may be attributed to more strict behavioral, religious and socio-cultural constraints on female premarital sex that are typically imposed on women in conservative societies. However, Gender difference in HBV seroprevalence has diminished since 2012 where no significant differences were observed between males and females. This observation emphasizes the positive effect of HBV vaccine implementation having a 98% to 100% established protection against hepatitis B (6).

With regard to HCV, data published by the Syrian MoH in 2004 revealed a 2.8% seropositivity of HCV in the general population, but a lower HCV prevalence in Syrian blood donors whose seropositivity fluctuated between 0.46 and 0.4% within the time frame (between 2003 and 2014) of that study (19). Similarly, data from Tartus Blood Transfusion Center reported a 0.48% HCV prevalence in 2017 (20). Interestingly, our data supports similar trends at Damascus University Blood Center with 0.52% overall prevalence rate.

Despite the analyses that indicate an average rate of 4% decrease in HCV antibodies prevalence per year in both the MENA region and Europe (39), no such decline was observed in Syrian blood donors across the 18 years of our study. Instead, our analyses of the data from Damascus University Blood Center revealed a significant decrease in HCV prevalence since 2018. This HCV decline could be attributed to the availability of internationally-recommended and low-cost pan-genotypic interferon-free (INF-free) direct-acting antiviral (DAA) regimen (sofosbuvir and daclatasvir) (40, 41), in addition to the increase in the total numbers of people planned and budgeted for treatment of HCV infection since 2017, as reported by the WHO Hepatitis Country Profile of Syria (41, 42). Furthermore, our findings indicated that the prevalence of HCV was significantly lower than that of HBV. That is probably due to the greater infectivity, longer survival and higher contagiousness of HBV risky sexual activities compared to HCV, which is transmitted mainly through transfusion of blood or blood products, intravenous drug abuse, and needle sharing (43). Donors in the age group of 18–25 years old were less likely to have HCV infection compared to other age groups. This outcome might be due to the increased risk of developing chronic HCV infection in patients of older age because of impaired immune systems and comorbidities (28, 44).

The scarcity and unreliability of data concerning HIV are major limitations that hinder accurate estimation of its prevalence in many of the MENA region countries, including Syria. Published statistics are often based on national authorities working in cooperation with the Joint United Nations Program on HIV/AIDS (UNAIDS) using mathematical modeling and prediction; this usually leads to an under-estimation of the HIV burden and dynamics in this region (45). Our study revealed an overall seroprevalence of HIV infection of 0.23%. Recently, Centers for Disease Control and Prevention (CDC) have addressed HIV prevalence among Syrian refugees and reported that medical screening of Syrian refugees who arrived in Texas between 2012 and 2016 proved low rates of HIV infection (0.8%) (13). Additionally, a retrospective study of 11,015 Syrian pregnant women in Turkey between 2012 and 2018 revealed a 0.03% seropositivity rate of anti-HIV (46). We attributed the differences between the aforementioned studies and our findings to the different demographic characteristics and size of the study populations.

The trend of HIV positivity rates was relatively fluctuating between 2004 and 2009, but showed a descending trend since 2011. Additionally, Damascus University Blood Center data showed a 70.37% decrease in HIV prevalence in donors from 2010 (0.27%) to 2011 (0.08%) (Table 1).

Global reports from 2012 showed a 29% decrease in HIV prevalence since 2010 among the general Syrian population (22, 40). Furthermore, premarital HIV testing was introduced in 2010 (25), which helped in monitoring HIV spread, in addition to national and WHO-supported programs and government commitment to the global provision of HIV prevention and elimination.

Generally, the prevalence of co-infections in our sample was low (0.008%), which may be due to the young age of our blood donor pool. The most frequently encountered co-infection was HBV-HCV (69.23%) followed by HBV-HIV (23.07%) and HCV-HIV (7.69%). No cases of a triple infection (HBV-HCV-HIV) was reported in this current study. Reports that documented multi-infections in the neighboring countries were relatively scarce, which limited the possibility of making any relevant comparison(s). However, previous literature attributed the majority of co-infection cases to HBV-HIV, followed by HBV-HCV, and then to HIV-HCV (47). Chaabna et al. reviewed the data belonging to HCV-HIV co-infections across the MENA region and showed a similar pattern in the neighboring Lebanon (7.7%) and Morocco (5.4%), but a higher prevalence of HCV-HIV in Tunisia (33.5%) (48).

Despite the relatively large sample size and the immense volume of data acquired during this study over 18 years, this work has some limitations. As a retrospective study, it was limited by the availability of some demographic data (such as the marital status, education level, and occupation) and HBV vaccination status. Additionally, the performed screening tests were serological rather than molecular tests (Nucleic Acid Test, NAT), meaning that they may underestimate the prevalence of TTVIs due to the presence of the “window period.” Besides, some chronically infected individuals actually have fluctuating HBV viremia, while others may show lack of detectable HBsAg due to replication deficiencies and viral genome mutations (49).

## 5. Conclusions

In this study, we evaluated the TTVI screening data of blood donors at Damascus University Blood Center over a period of 18 years spanning 7 years (2004–2010) prior to the armed conflict and 11 years (2011–2021) during the Syrian war. Our findings revealed a significant regression in HBV and HIV prevalence from 2011 to 2021, and therefore support the perceived effectiveness of the hepatitis B vaccine that was mandated in 1993 in reducing the spread of the disease. Although TTVIs prevalence among blood donors may underestimate prevalence in the general populations due to self-selection of blood donors or the effectiveness of pre-donation screening; this and similar studies still reflect general aspects and trends of TTVIs spread in the general population especially when governmental updates on prevalence are missing. Future studies compiling and analyzing data from other national blood banks across the country will unquestionably provide a more accurate and comprehensive representation of the status of TTVI prevalence in the demographically and culturally diverse Syrian population.

## Data availability statement

The original contributions presented in the study are included in the article/Supplementary material, further inquiries can be directed to the corresponding author.

## Author contributions

AA: data organization, resources, and validation. MJR: software, formal analysis, and co-writing—original draft. AY: investigation, co-writing—original draft, and visualization. SM and AF: formal analysis and co-writing—original draft. LY: conceptualization, writing—review and editing, supervision, and project administration. All authors contributed to the article and approved the submitted version.
