# Supplementary material for: Seroprevalence and trends of hepatitis B virus, hepatitis C virus and human immunodeficiency virus in Syrian blood donors at Damascus University Blood Center between 2004 and 2021
Source: Front Public Health. 2023 Jun 1;11:1174638. doi: 10.3389/fpubh.2023.1174638 (PMC10267398; doi:10.3389/fpubh.2023.1174638)
Supplement: Supplementary file 1 [file Image_1.pdf]

Supplementary Material

Seroprevalence and Trends of Hepatitis B Virus, Hepatitis C Virus and Human Immunodeficiency Virus in Syrian Blood Donors at Damascus University Blood Center between 2004 and 2021

Alia Alassad[1], Mhd Jawad Al Rahwanji[2,3], Amal Yousfan[4,5], Sally Al-Moualem[6], Arwa Farhat[6], Lama A. Youssef[7*]

*** Correspondence:**

Lama A. Youssef: lama.youssef@damascusuniversity.edu.sy **or** ylama@hotmail.com

1 Supplementary Figures and Tables

1.1 Supplementary Figures

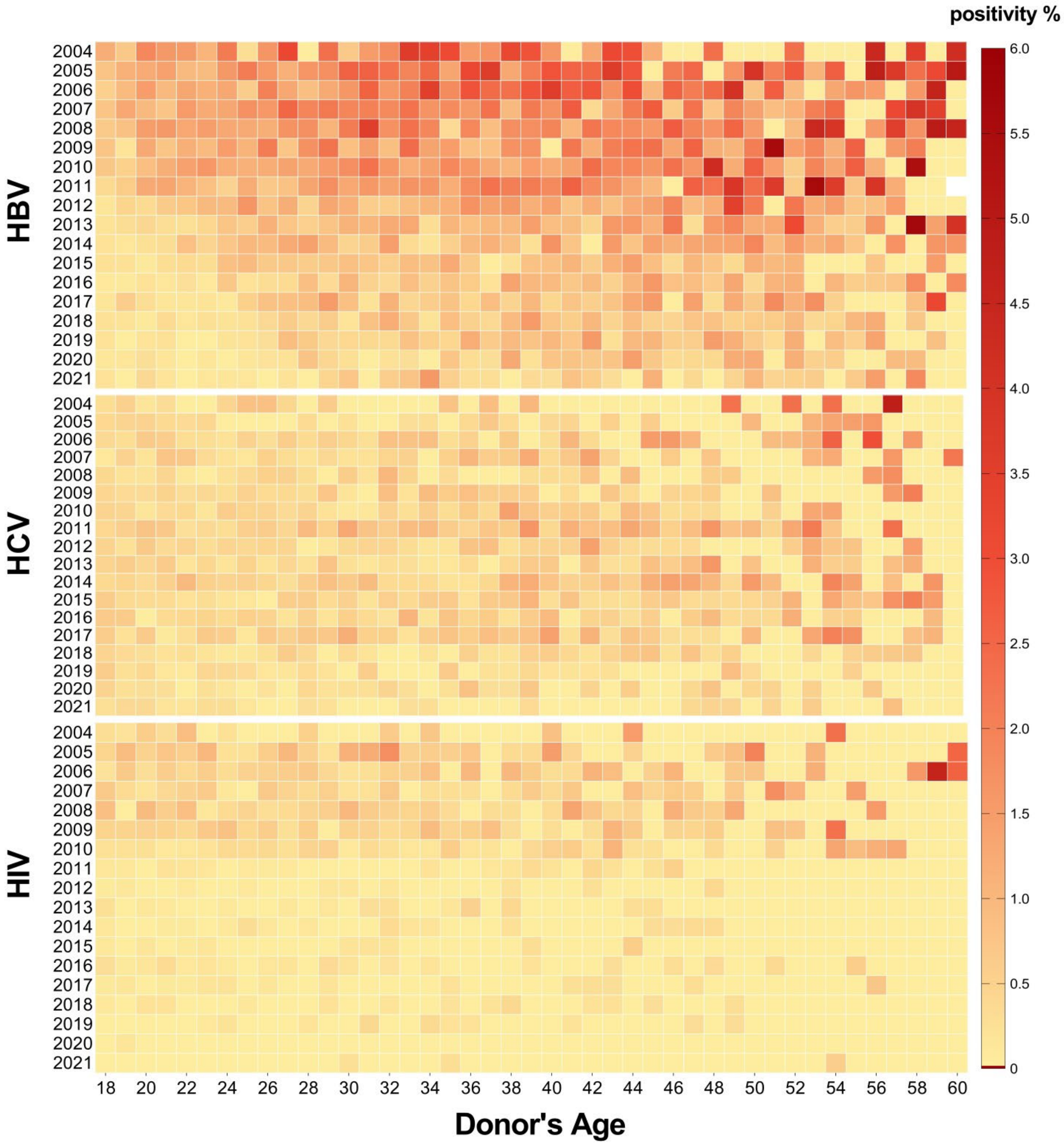

Supplementary Figure 1. Heatmaps of HBV, HCV and HIV seropositivities in donors 18 to 60 years old for the 18-year study period spanning the years 2004 to 2021. HBV positivity depicted a diagonal decline starting in 2012 (i.e., 18 years after introducing the HBV vaccine to the National Vaccination Program), and the years that followed in young donors who were born after 1993. The HCV infections remained the same in terms of prevalence throughout the years; whereas the seropositivity of HIV infections dropped starting in 2011 and across all ages.
